# Protein secondary structure prediction using a small training set (compact model) combined with a Complex-valued neural network approach

**DOI:** 10.1186/s12859-016-1209-0

**Published:** 2016-09-13

**Authors:** Shamima Rashid, Saras Saraswathi, Andrzej Kloczkowski, Suresh Sundaram, Andrzej Kolinski

**Affiliations:** 1 School of Computer Science and Engineering, Nanyang Technological University, 50 Nanyang Ave, Singapore, 639798 Singapore; 2 Battelle Center for Mathematical Medicine, The Research Institute at Nationwide Children’s Hospital, 700 Children’s Drive, Columbus, USA; 3 Sidra Medical and Research Center, Al Dafna, Doha, Qatar; 4 Department of Paediatrics, College of Medicine, The Ohio State University, 370 W. 9th Avenue, Columbus, USA; 5 Laboratory of Theory of Biopolymers, Faculty of Chemistry, University of Warsaw, Pasteura 1, Warsaw, 02-093 Poland

**Keywords:** Secondary structure prediction, Heuristics, Complex-valued relaxation network, Inhibitor peptides, Efficient learning, Protein structure, Compact model

## Abstract

**Background:**

Protein secondary structure prediction (SSP) has been an area of intense research interest. Despite advances in recent methods conducted on large datasets, the estimated upper limit accuracy is yet to be reached. Since the predictions of SSP methods are applied as input to higher-level structure prediction pipelines, even small errors may have large perturbations in final models. Previous works relied on cross validation as an estimate of classifier accuracy. However, training on large numbers of protein chains compromises the classifier ability to generalize to new sequences. This prompts a novel approach to training and an investigation into the possible structural factors that lead to poor predictions.

Here, a small group of 55 proteins termed the compact model is selected from the CB513 dataset using a heuristics-based approach. In a prior work, all sequences were represented as probability matrices of residues adopting each of Helix, Sheet and Coil states, based on energy calculations using the **C**-**A**lpha, C-**B**eta, **S**ide-chain (CABS) algorithm. The functional relationship between the conformational energies computed with CABS force-field and residue states is approximated using a classifier termed the Fully Complex-valued Relaxation Network (FCRN). The FCRN is trained with the compact model proteins.

**Results:**

The performance of the compact model is compared with traditional cross-validated accuracies and blind-tested on a dataset of G Switch proteins, obtaining accuracies of ∼81 %. The model demonstrates better results when compared to several techniques in the literature. A comparative case study of the worst performing chain identifies hydrogen bond contacts that lead to Coil ⇔ Sheet misclassifications. Overall, mispredicted Coil residues have a higher propensity to participate in backbone hydrogen bonding than correctly predicted Coils.

**Conclusions:**

The implications of these findings are: (i) the choice of training proteins is important in preserving the generalization of a classifier to predict new sequences accurately and (ii) SSP techniques sensitive in distinguishing between backbone hydrogen bonding and side-chain or water-mediated hydrogen bonding might be needed in the reduction of Coil ⇔ Sheet misclassifications.

**Electronic supplementary material:**

The online version of this article (doi:10.1186/s12859-016-1209-0) contains supplementary material, which is available to authorized users.

## Background

The earliest models of protein secondary structure were proposed by Pauling and Corey who predicted that the polypeptide backbone contains regular hydrogen bonded geometry, forming *α*- helices and *β*-sheets [1, 2]. The subsequent deposition of structures into public databases aided growth of methods predicting structures from protein sequences. Although the number of structures in the Protein Data Bank (PDB) is growing at an exponential rate due to advances in experimental techniques, the number of protein sequences remains far higher. The NCBI *RefSeq* database [3] contains 47 million protein sequences and the PDB, ∼110,000 structures (including redundancy) as of April 2016. Therefore, the computational prediction of protein structures from sequences still remains a powerful complement to experimental techniques. Protein Secondary Structure Prediction (SSP), often an intermediate step in the prediction of tertiary structures has been of great interest for several decades. Since structures are more conserved than sequences, accurate secondary structure predictions can aid multiple sequence alignments and threading to detect homologous structures, amongst other applications [4]. The existing SSP methods are briefly summarized by developments that led to increases in accuracy and grouped by algorithms employed.

The GOR technique pioneered the use of an entropy function employing residue frequencies garnered from proteins databases [5]. Later, the development of a sliding window scheme and the calculation of pair wise propensities (rather single residue frequencies) resulted in an accuracy of 64.4 % [6]. Subsequent developments include combining the GOR technique with evolutionary information [7, 8] and the incorporation of the GOR technique with a fragment mining method [9, 10]. The PHD method employed multiple sequence alignments (MSA) as input in combination with a two level neural network predictor [11], increasing the accuracy to 72 %. The representation of an input sequence as a profile matrix obtained from PSI-BLAST [12] derived position specific scoring matrices (PSSM) was pioneered by PSIPRED, improving the accuracy up to 76 % [13]. Most techniques now employ PSSM (either solely or in combination with other protein properties) as input to machine-learning algorithms. The neural network based methods [14–21] have performed better than other algorithms in recent large scale reviews that compared performance on up to 2000 protein chains [22, 23]. Recently, more neural network based secondary structure predictors have been developed, such as the employment of a general framework for prediction [24], and the incorporation of context-dependent scores that account for residue interactions in addition to the PSSM [25]. Besides the neural networks, other methods use support vector machines (SVM) [26, 27] or hidden Markov models [28–30]. Detailed reviews of SSP methods are available in [4, 31]. Current accuracies tested on nearly 2000 chains yield up to 82 % [22]. In the machine learning literature, neural networks employed in combination with SVM obtained an accuracy of 85.6 % on the CB513 dataset [32]. Apart from the accuracies given in reviews, most of the literature reports accuracy based on machine-learning models employing k-fold cross-validation and does not provide insight to underlying structural reasons for poor performance.

### The compact model

The classical view adopted in developing SSP methods is that a large number of training proteins are necessary, because the more proteins the classifier is trained on, the better the chances of predicting an unseen protein sequence e.g. [18, 33]. This involved large numbers of training sequences. For example, SPINE employed 10-fold cross validation on 2640 protein chains and OSS-HMM employed four-fold cross-validation on approximately 3000 chains [18, 29]. Cross-validated accuracies prevent overestimation of the prediction ability. In most of the protein SSP methods, a large number of protein chains (of at least a thousand) have been used to train the methods. Smaller numbers by comparison, (in the hundreds) have been used to test them. The ratio of train to test chains is 8:1, for YASPIN [28] and ∼5:1 for SPINE and SSPro [14]. However, the exposure to large numbers of similar training proteins or chains may result in over training and thereby influence the generalization ability when tested against new sequences.

A question arises on the possible existence of a smaller number of proteins which are sufficient to build an SSP model that achieves a similar or better performance. Despite the high accuracies described, the theoretical upper limit for the SSP problem, estimated at 88–90 %, has not been reached [34, 35]. Moreover, some protein sequences are inherently difficult to predict and the reasons behind, unclear. An advantage of a compact model is that the number of folds used in training is small and often distinct from the testing proteins. Subsequently, one could add proteins whose predictions are unsatisfactory, into the compact model. This may identify poorly performing folds, or other structural features which are difficult to predict correctly by existing feature encoding techniques or classifiers. This motivates our search for a new training model for the SSP problem.

The goal of this paper is to locate a small group of proteins from the proposed dataset, such that training the classifier on them maintains similar accuracies to cross-validation, yet retains its ability to generalize to new proteins. Such a small group of training proteins is termed as the ‘compact model’, representing a step towards an efficient learning model that prevents over fitting. Here, the CB513 dataset [36] is used to develop the compact model and a dataset of G Switch proteins (GSW25) [37] is used for validation. A feature encoding based on computed energy potentials is used to represent protein residues as features. The energy potential based features are employed with a fully complex-valued relaxation network (FCRN) classifier to predict secondary structures [38]. The compact model employed with the FCRN provides a similar performance compared to cross-validated approaches commonly adopted in the literature, despite using a much smaller number of training chains. The performance is also compared with several existing SSP methods for the GSW25 dataset.

Using the compact model, the effect of protein structural characteristics on prediction accuracies is further examined. The Q _3_ accuracies across Structural Classification of Proteins (SCOP) classes [39] are compared, revealing classes with poor Q _3_. For some chains in these poor performing SCOP classes, the accuracy remains low (below 70 %) even if they were to be included as training proteins, or even if tested against other techniques in the literature. The possible structural reasons behind the persistent poor performance were investigated, but it was difficult to attribute the source (e.g. mild distortions induced by buried metal ligands). However, a detailed case study of the porcine trypsin inhibitor (the worst performing chain) highlights the possible significance of water-mediated vs. peptide-backbone hydrogen bonded contacts towards the accuracy.

The remaining of the paper is organized as follows. The *Methods* section describes the datasets, feature encoding of the residues (based on energy potentials) and the architecture and learning algorithm of the FCRN classifier. Next, the heuristics-based approach is presented to obtain the compact model. Section *Performance of the compact model* investigates the performance of the compact model compared with cross-validation in two datasets: the remainder of the CB513 dataset and on GSW25. The section *Case study of two inhibitors* presents the case study in which the trypsin inhibitor is compared with the inhibitor of the cAMP dependent protein kinase. The differences in the structural environments of Coil residues in these inhibitors are discussed with respect to the accuracy obtained. The main findings of the work are summarized in *Conclusions*.

## Methods

### Datasets

**CB513** The benchmarked CB513 dataset developed by Cuff and Barton is used [36]. 128 chains were further removed from this set by Saraswathi et al., [37], to avoid homology with CATH structural templates used to generate energy potentials (see *CABS-Algorithm based Vector Encoding of Residues*). The resultant set has 385 proteins comprising 63,079 residues. The composition is approximately 35 % helices, 23 % strands and 42 % coils. Here, the first and last four residues of each chain are excluded in obtaining the compact model (see *Development of compact model*), giving a final set containing 59,999 residues which comprise 35.3 % helices, 23.2 % strands and 41.4 % coils, respectively.

**G Switch Proteins (GSW25)** This dataset was generated during our previous work on secondary structure prediction [37]. It contains 25 protein chains derived from the G _*A*_ and G _*B*_ domains of the *Streptococcus* G protein [40, 41]. The G _*A*_ and G _*B*_ domains bind human serum albumin and Immunoglobulin G (IgG), respectively. There are two folds present: a 3 *α* fold and 4 *β* + *α* fold corresponding to the G _*A*_ and G _*B*_ domains, respectively. A series of mutation experiments investigated the role of residues in specifying one fold over the other, hence the term ‘switch’ [42].

The dataset contains similar sequences. However, it is strictly used for blind testing and not used in model development. The sequence identities between CB513 and GSW25 are less than 25 % as checked with the PISCES sequence culling server [43]. The compact model obtained does not contain either the *β*-Grasp ubiquitin-like or albumin binding domain-like folds, corresponding to G _*A*_ and G _*B*_ domains according to SCOP classification [39]. In this set, 12 chains belong to G _*A*_ and 13 chains to G _*B*_, with each chain being 56 residues long. The total number of residues is 1400 and comprises 52 % helix, 39 % strand and 9 % coil respectively. The sequences are available in Additional file 1: Table S1.

The secondary structure assignments were done using DSSP [44]. The eight to three state reduction is performed as in other works [18, 37]. States H, G, I (*α*,3_10_,*π* helices) were reduced to Helix (H) and states E, B (extended, single residue *β*-strands) to Sheet (E). States T, S and blanks (*β*-turn, bend, loops and irregular structures) were reduced to Coil (C).

### CABS-algorithm based vector encoding of residues

We used knowledge-based statistical potentials to encode amino acid residues as vectors instead of using PSSM. This data was generated during our previous work [37] on secondary structure prediction. Originally these potentials were derived for coarse grained models (CABS- **C**-**A**lpha, C-**B**eta and **S**ide-chains) of protein structure. CABS could be a very efficient tool for modeling of protein structure [45], protein dynamics [46] and protein docking [47]. The force-field of CABS model has been derived using careful analysis of structural regularities seen in a representative set of high resolution crystallographic structures [48].

This force-field consist of unique context-dependent potentials, that encode sequence independent protein-like conformational preferences and context-dependent contact potentials for the coarse-grained representation of the side chains. The side chain contact potentials depend on the local geometry of the main chain (secondary structure) and on the mutual orientation of the interacting side chains. A detailed description of the implementation of CABS-based potentials in our threading procedures could be found in [37]. It should be pointed out, that use of these CABS-based statistical potentials (derived for various complete protein structures, and therefore accounting for structural properties of long range sequence fragments) opens the possibility for effective use of relatively short windows size for the target-template comparisons. Another point to note is the fact that the CABS force-field encodes properly averaged structural regularities seen in the huge collection of known protein structures. Since such an encoding incorporates proper averages for large numbers of known protein structures, the use of a small training set does not reduce the predictive strength of the proposed method for rapid secondary structure prediction.

**Fig. 1:**
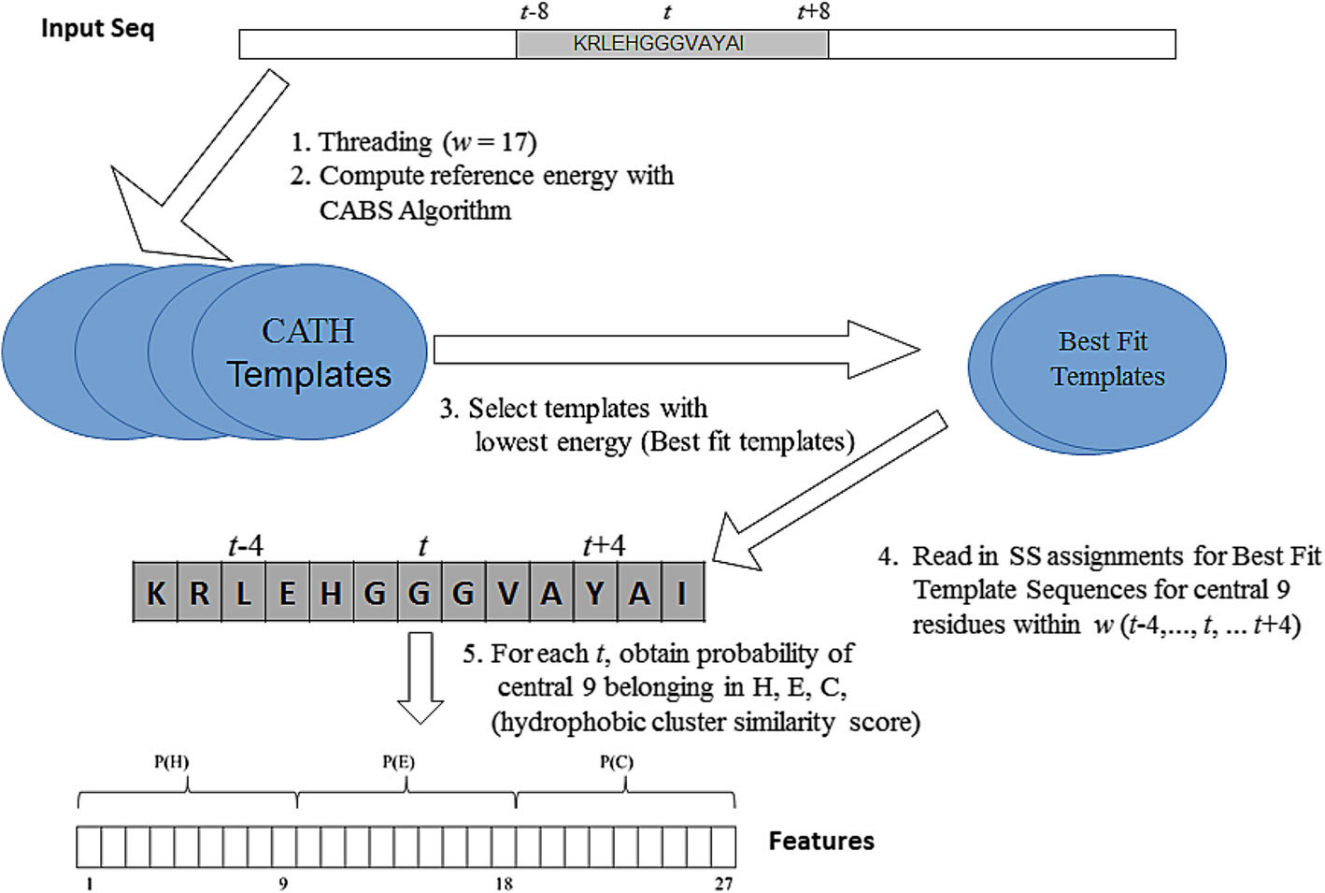
Representation of features. A target residue, *t* in the input sequence is represented as a 27-dimensional feature vector. The input sequence is read in a sliding window (*w*) of 17 residues (*grey*). The central residue (*t*) and several of its neighbours to the left and right are shown. CATH templates were previously assigned SS using DSSP. Target to template threading was done using *w* = 17 and the reference energy computed with the CABS-algorithm. The SS are read in from best fit template sequences that have the lowest energy for the central 9 residues within *w*. Since multiple SS assignments will be available for a residue, *t* and its neighbours from from templates, the probability of each SS state is computed using a hydrophobic cluster similarity score. P(H), P(E) and P(C) denote probabilities of *t* and its four neighbours to the left and right, adopting Helix, Sheet and Coil structures respectively. CATH templates are homology removed and independent with respect to the CB513 dataset

A target residue was encoded as a vector of 27 features, with the first 9 containing its propensity to form Helix (H), the next 9 its propensity to form Sheet (E) and the last 9, its propensity to form Coil (C) structures (see Fig. 1). The process of encoding was described in [37] and is repeated here.

## Removal of highly similar targets

In this stage, target sequences that have a high similarity to templates were removed to ensure that the predicted CB513 sequences are independent of the templates used. Therefore the accuracies reported may be attributed to other factors such as the CABS- algorithm, training or machine-learning techniques used, rather than an existing structural knowledge.

A library of CATH [49] structural templates was downloaded and Needleman-Wunsch [50] global alignment of templates to CB513 target sequences was performed. There were 1000 template sequences and 513 target sequences, resulting in 513000 pairwise alignments. Of these alignments, 97 % had similarity scores in the range of 10 to 18 % and the remaining 3 % contained up to 70 % sequence similarity (see Figure S7 in [37]). However, only 422 CATH templates could be used due to computational resource concerns and PDB file errors. Structural similarities between targets and templates were removed by querying target names against Homology-derived Secondary Structure of Proteins (HSSP) [51] data for template structures. After removal of sequence or structural similarities, 422 CATH structural templates and 385 proteins from CB513 were obtained. The DSSP secondary structure assignments were performed for these templates. Contact maps were next computed for the heavy atoms C, O and N with a distance cutoff of 4.5 Å.

## Threading and computation of reference energy

Each target sequence was then threaded onto each template structure using a sliding window of size 17 and the reference energy computed using the CABS-algorithm. The reference energy takes the (i) short-range contacts, (ii) long-range contacts and (iii) hydrophobic/hydrophilic residue matching into account, weighted 2.0 :0.5 :0.8, respectively [37]. For short range residues, reference energies depend on molecular geometry and chemical properties of neighbours up to 4 residues apart. For long-range interactions, a contact energy term is added if aligned residues are interacting according the contact maps generated in the previous stage. The best matching template residue is selected using a scoring function (unpublished). The lowest energy (best fit) residues are retained.

The DSSP secondary structure assignments from the best fitting template sequences are read in, but this was done only for the 9 central residues in the window of 17. The probability of the 9 central residues adopting each of the three states Helix, Sheet or Coil is derived using a hydrophobic cluster similarity based method [52]. Figure 1 illustrates the representation of an amino acid residue from an input sequence as a vector of 27 features in terms of probabilities of adopting each of the three secondary structures H, E or C.

It is emphasized that the secondary structures of targets are not used in the derivation of features. However, since target-template threading of sequences was performed, the method indirectly incorporates structural information from the best matching templates. A complete description of the generation of the 27 features for a given target residue is available in [37]. These 27 features serve as input to the classifier that is described next.

### Fully complex valued relaxation network (FCRN)

The FCRN is a complex-valued neural network classifier that uses a complex plane as its decision boundary. In comparison with real-valued neurons, the orthogonal decision boundaries afforded by the complex plane can result in more computational power [53]. Recently the FCRN was employed to obtain a five-fold cross-validated predictive accuracy of 82 % on the CB513 dataset [54]. The input and architecture of the classifier are described briefly.

Let a residue *t* be represented by **x**^*t*^ where **x** is the vector containing 27 probability values pertaining to the three secondary structure states H, E or C. **x**^*t*^ was normalized to lie between -1 to +1 using the formula $2\times [\frac {\mathbf {x}^{t} - min({\mathbf {x}^{t})}}{ max({\mathbf {x}^{t})-min({\mathbf {x}^{t})}}}]$. The normalized **x**^*t*^ values were mapped to the complex plane using a circular transformation. The complex-valued input representing a residue is denoted by **z**^*t*^ and coded class labels **y**^*t*^ denote the complex-valued output.

**Fig. 2:**
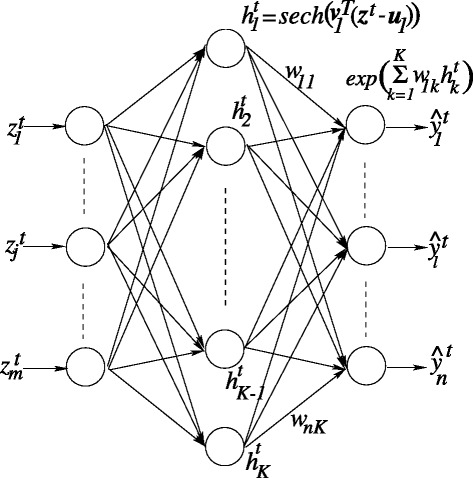
The Architecture of FCRN. The FCRN consists of a first layer of *m* input neurons, a second layer of *K* hidden neurons and a third layer of *n* output neurons. For the SS prediction problem presented in this work, *m*=27, *n*=3 and *K* is allowed to vary. The hyperbolic secant (*sech*) activation function computes the hidden response (${h^{t}_{l}}$) and the predicted output $\widehat {y}_{l}^{t}$ is given by the exponential function. *w*_*nK*_ represents the weight connecting the *Kth* hidden neuron to the *nth* output neuron

FCRN architecture is similar to three layered real networks as shown in Fig. 2.

However, the neurons employ the Complex plane. The first layer contains *m* input neurons that perform the circular transformation that map real-valued input features onto the complex plane. The second layer employs *K* hidden neurons employing the hyperbolic secant (*sech*) activation function. The output layer contains *n* neurons employing an exponential activation function. The predicted output is given by

1 $$\begin{array}{@{}rcl@{}} \widehat{y}_{l}^{t} &=& exp\left(\sum\limits_{k=1}^{K} w_{lk}{h_{k}^{t}}\right) \end{array} $$

Here, ${h_{k}^{t}}$ is the hidden response and *w*_*lk*_ the weight connecting the *k*^*th*^ hidden unit and *l*^*th*^ output unit. The algorithm uses projection based learning where optimal weights are analytically obtained by minimizing an error function that accounts for both magnitude and phase of the error. A different choice of classifier could potentially be used to locate a small training set. However, since it has been shown in the literature that complex-valued neural networks are computationally powerful due to their inherent orthogonal decision boundary, here the FCRN was employed to select proteins of the compact model and to predict secondary structures. Complete details of the learning algorithm are available in [38].

### Accuracy measures

The scores used to evaluate the predicted structures are the Q _3_ which measures single residue accuracy (correctly predicted residues over total residues), as well as the segment overlap scores SOV _*H*_, SOV _*E*_ and SOV _*C*_, which measure the extent of overlap between native and predicted secondary structure segments for Helix (H), Sheet (E) and Coil (C) states, respectively. The overall segment overlap for the three states is denoted by SOV. The partial accuracies of single states, Q _*H*_, Q _*E*_ and Q _*C*_, which measure correctly predicted residues of each state over the total number of residues in that state, is also computed.

All segment overlap scores follow the definition in [55] and were calculated with Zemla’s program. The per-class Matthew’s Correlation Coefficient (MCC) follows the definition in [23]. The class-wise MCC _*j*_ with *j*∈*H*,*E*,*C* is obtained by

$${{} {\begin{aligned} MCC_{j} =\frac{TP\times TN-FP\times FN}{\sqrt{(TP+FP)\times (TP+FN)\times(TN+FP)\times(TN+FN)}} \end{aligned}}} $$

Here, TP denotes true positive (number of correctly predicted positives in that class, e.g. native helices which are predicted as helices; FP denotes false positive (no. of negative natives predicted as positives), i.e. sheets and coils predicted as helices); TN denotes true negative (number of negative natives predicted negative, i.e. no. of non-helix residues predicted as either sheets or coils); FN denotes false negative (number of native positives predicted negative, i.e. no. of helices misclassified as sheets and coils). Similar definitions follow for Sheets and Coils.

### Development of compact model

The feature extraction procedure uses a sliding window of size 9 (see Section *CABS-algorithm based vector encoding of residues*), resulting in lack of neighbouring residues for the first and last four residues in a sequence. Since they lack adequate information, the first and last four residues were not included in the development of the compact model. Besides, the termini of a sequence are subject to high flexibility resulting from physical pressures; for instance the translated protein needs to move through Golgi apparatus. Regardless of sequence, flexible structures may be highly preferred. This could introduce much variation in the sequence to structure relationship that is being estimated by the classifier, prompting for the decision to model them in a separate work. Here, it was of interest to first establish that training with a small group of proteins is viable.

**Fig. 3:**
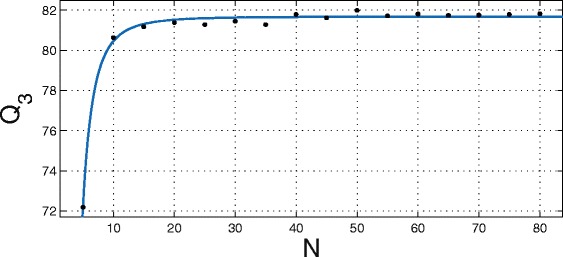
Q _3_ vs no. of training sequences (N). The accuracy achieved by FCRN as a function of increasing N is shown. Highest Q _3_ is observed at 82 % for 50 sequences. Maximum allowed hidden neurons = 100

Since the number of training proteins required to achieve the maximum Q _3_ on the dataset is unknown, it was first estimated by randomized trials. The 385 proteins derived from CB513 were numbered from 1 to 385 and the uniformly distributed *rand* function from MATLAB was used to generate unique random numbers within this range. At each trial, 5 sequences were added to the training set and the Q _3_ accuracy (for that particular set) was obtained by testing on the remainder. The number of hidden neurons was allowed to vary but capped at a maximum of 100. The Q _3_ scores have been shown as a function of increasing the number of training proteins in Fig. 3.

The Q _3_ clearly peaks at 82 % for 50 proteins, indicating that beyond this number, the addition of new proteins contributes very little to the overall accuracy and even worsens it slightly at 81.72 %. All trials were conducted using MATLAB R2012b running on a 3.6 GHz machine with 8GB RAM on a Windows 7 platform.

**Fig. 4:**
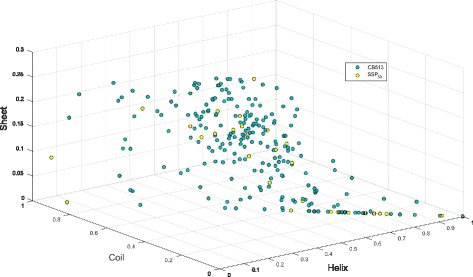
Plot of CB513 proteins by their secondary structure content. *One circle* represents a single protein sequence. SSP _50_ proteins are represented as *yellow circles* while the remainder of the CB513 dataset are *green circles*. The compact model, SSP _55_ proteins are spread out in a similar fashion to the SSP _50_ proteins shown here. Axes show the proportion of Helix, Coil and Sheet residues divided by the sequence length. For instance, a hypothetical 30 residue protein comprised of only Helix residues, would be represented at the *bottom-right most* corner of the plot

**Heuristics-based selection of best set:** Using 50 as an approximate guideline of the number of proteins needed, various protein sets were selected such that accuracies achieved are similar to cross-validation scores reported in the literature (e.g. about 80 %). These training sets are:

1. SSP _*sampled*_. Randomly selected 50 proteins (∼7000 residues), distinct from the training sets shown in Fig. 3.
2. SSP _*balanced*_. Randomly selected residues (∼8000) containing equal numbers from each of H, E, C states.
3. SSP _50_. 50 proteins (∼8000 residues) selected by visualizing CB513 proteins according to H, E, C ratios. Proteins with varying ratios of H, E, C structures were chosen such that representatives were picked over the secondary structure space populated by the dataset (see Fig 4).

Tests on the remainder of the CB513 dataset indicated only a slight difference in accuracy between the above training sets, with Q _3_ values hovering at ∼81 %. The sets of training sequences from Q _3_ vs. N experiments (Fig. 3) as well as the three sets listed above were tested against GSW25, revealing a group of 55 proteins that give the best results. The 55 proteins have been presented in Additional file 1: Table S2. These 55 proteins are termed the compact model. A similar technique could be applied on other datasets and is described here as follows.

**Fig. 5:**
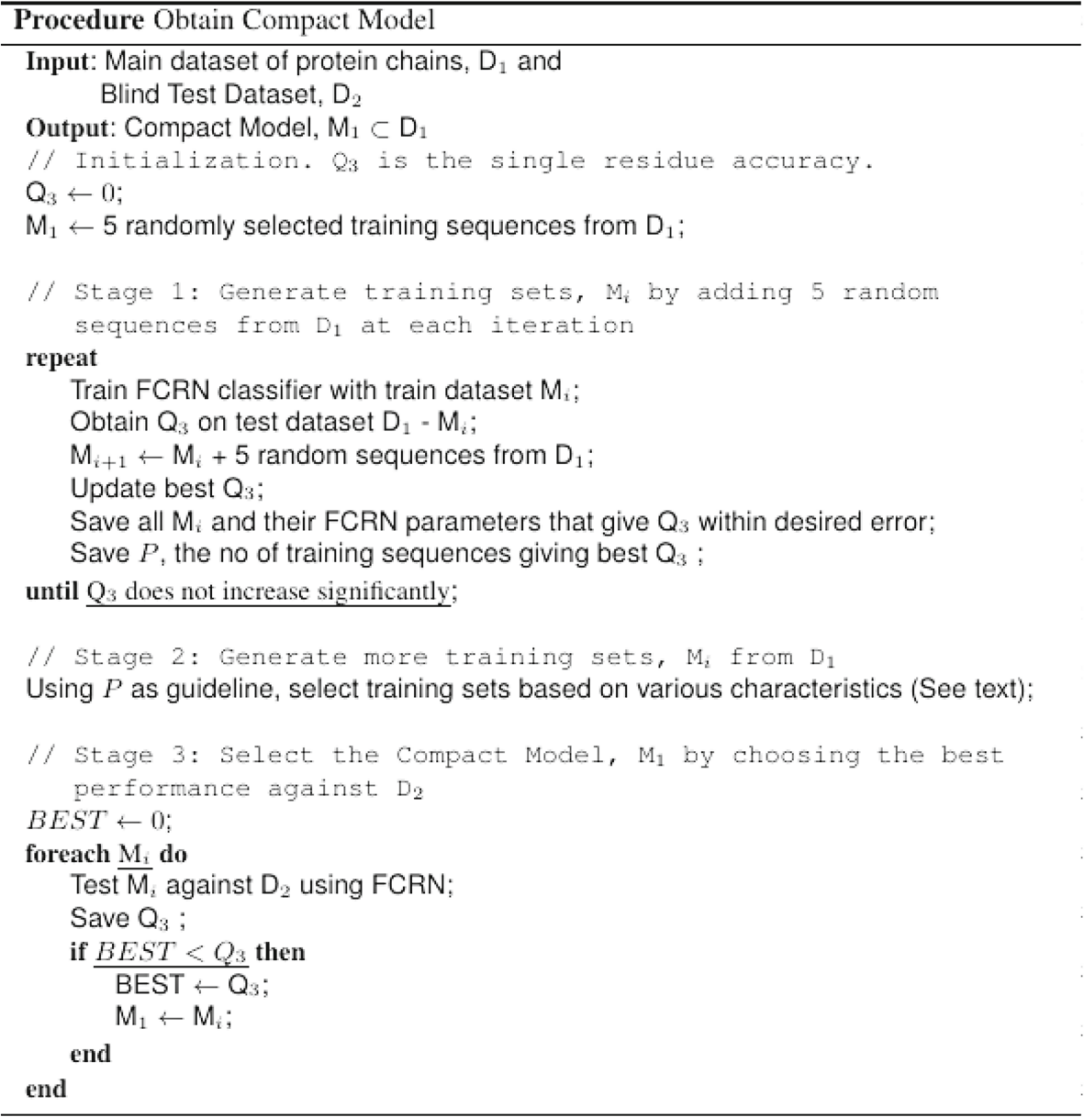
Procedure obtain compact model

The development of a compact model follows three stages. First, the number of training proteins, *P* needed to achieve a desired accuracy on a given dataset, is estimated by randomly adding chains to an initial small training set and monitoring the effect on Q _3_. This first stage also necessarily gives several randomly selected training sets of varying sizes. Second, *P* is used as a guideline for the construction of additional, training sets that are selected according to certain characteristics such as the balance of classes within chains (described under the heading ‘Heuristics-based Selection of Best Set’). Here, other randomly selected proteins may also form a training set. Other training sets of interest may also be constructed here. In the third stage, the resultant training sets from stages one and two are tested against an unknown dataset. The best performing set of these, is termed the compact model. Procedure ‘Obtain Compact Model’ given in Fig. 5 shows the stages described.

## Results and discussion

### Performance of the compact model

First, a five-fold cross-validated study, similar to other methods reported in the literature was conducted to serve as a basis for comparison for the compact model. The 385 proteins were divided into 5 partitions by random selection. Each partition contained 77 sequences and was used once for test, with the rest for training. Any single protein served only once as a test protein, ensuring that final results reflected a full training on the dataset.

The compact model of 55 training proteins is denoted SSP _55_ and the cross-validation model, SSP _*CV*_. For SSP _55_, the remaining 330 proteins containing 51,634 residues served as the test set. For a fair comparison, SSP _*CV*_ results for these same 330 test proteins were considered. The FCRN was separately trained with parameters from both models and was allowed to have a maximum of 100 hidden neurons. Train and test times averaged for 100 residues were 4 min and 0.3 s, respectively on a 3.6 GHz processor with 8G RAM. Results are shown in Table 1. The performance of SSP _55_ was extremely close to that of SSP _*CV*_ across most predictive scores as well as the Matthew’s correlation coefficients (MCC). Further discussion follows.

**Table 1:** Results on CB513 (51,634 residues)

Model	Observed *j*		Predicted *j*		Q _*j*_ (%)	Q _3_ (%)	SOV _*j*_ (%)	SOV (%)	MCC _*j*_
		H	E	C					
	H	16469	48	1840	89.72		83.14		0.82
SSP _*CV*_	E	92	8804	2955	74.29	82.03	72.24	79.46	0.71
	C	2313	2032	17081	79.73		75.46		0.64
	H	16333	62	1962	88.98		82.19		0.81
SSP _55_	E	87	9001	2763	75.96	81.72	73.43	78.93	0.71
	C	2288	2279	16859	78.69		74.5		0.63

The Q _3_ values for SSP _55_ and SSP _*CV*_ were 81.72 % and 82.03 % respectively. This is a small difference of 0.31 % which amounts to 160 residues in the present study. As reported in earlier studies [18, 22] it was easiest to predict Helix residues followed by Coil and Sheet for both the SSP _55_ and SSP _*CV*_ models. The Q _*H*_, Q _*E*_ and Q _*C*_ values were 89.72 %, 74.29 %, 79.73 % respectively under the SSP _*CV*_ model and 88.98 %, 75.96 % and 78.69 % under the SSP _55_ model. SSP _*CV*_ training predicted Helix and Coil residues better at about 1 %. The SSP _55_ model predicted Sheet residues better by 1.7 %.

The SOV score indicates SSP _*CV*_ predicted overall segments better by a half percentage point than SSP _55_. SSP _55_ predicted the strand segments better by 1.2 % with an SOV _*E*_ of 73.43 % vs. 72.24 % obtained by SSP _*CV*_. Similar findings were made when results of all 385 proteins (i.e. including training) were considered.

Since the results between both models were close, statistical tests were conducted to examine if the Q _3_ and SOV scores obtained per sequence were significantly different under the two models. For SSP _*CV*_, the scores used were averages of 5 partitions. First, the Shapiro-Wilk test [56] was conducted to detect if the scores are normally distributed. P values for both measures (<< 10 ^−5^) indicated that neither was normal at an *α*=0.05 level of significance. The non-parametric Wilcoxon signed-rank test [57] was next used to determine if paired values per sequence were significantly different. The P-values obtained for the Q _3_ and SOV measures were 0.0012 and 0.015, indicating that SSP _*CV*_ is better at a significance level of *α*=0.05.

It was expected that a smaller training set of 55 training proteins would give lower accuracies. However, the scores achieved were extremely close to those obtained from the larger training model (SSP _*CV*_). It is therefore remarkable that the increase in accuracy afforded by 5 times the number of proteins is less than half a percentage point for the Q _3_ score. SPINE reported seemingly different findings to those here [18]. A drop in Q _3_ of up to 4 percentage points was reported when smaller datasets were used in training. Other than the training sets, the accuracy achieved depends on factors like the choice of classifier and the type of feature encoding used. The latter two were different from the work here and could be a reason for the different conclusions.

It is further unknown if the sequence to structure information learnt by the network depends on entire proteins or if residue-based selection could show a comparable performance. In theory, if secondary structure involves mainly local interactions, residue-based training selections should yield comparable predictive accuracies. Since each amino acid residue is often encoded as a feature vector representing some properties of its sequential neighbours in a sliding window scheme, one could presume that local interactions are captured and that it is possible to randomly select residues for training rather than entire proteins. In 5-fold cross-validation experiments conducted previously, in which the partitions were created based on randomly selected residues rather than proteins, a Q _3_ score of 81.7 % was achieved [54]. However, training based on residues was found to improve Sheet prediction at the expense of the Coil class. The SSP _*Balanced*_ model was also created by selection of residues, but despite a high performance for sheet (Q _*E*_ = 83.83 %), the model gave a considerably lower accuracy for the Coil residues at 71.42 %.

A separate experiment was also conducted in which the first and last four residues of the 55 proteins of the compact model were included (see Section *Development of compact model* for reasons of exclusion). The Q _3_ obtained by the compact model was 81.5 % on 54,274 test residues, which indicates that a slight depreciation in performance (0.21 %) had been observed.

Results here suggest that most of the information relating to the structural folds present in CB513 is captured by the SSP _55_. Otherwise, the accuracies would have been much lower than expected with merely 55 training proteins.

### Effect of SCOP classes on accuracy

The composition of the CB513 dataset based on the Structural Classification of Proteins (SCOP) [39] classes was analysed to determine what effect structural classes have on the predictive accuracy. Effort was made to match CB513 sequences to sequences in PDB files derived from ATOM records. All 385 proteins were matched with current PDB structures with corresponding PDB identifiers and chains except for two of them. In some cases, obsolete PDB entries had to be kept to maintain sequence matches, but the IDs of superseding structures were also noted (385 proteins with PDB and SCOP identifiers is available on request). Using PDB identifiers, corresponding SCOP domains were assigned from parseable files of database version SCOPe 2.03. Sequences of the domains were also matched with the 385 proteins from CB513. For a majority of proteins, the sequences of the SCOP domains matched the CB513 sequences. The rest had partial or gapped matches, likely due to updated versions of defined domains for older structures. For such cases the corresponding domains were nevertheless assigned as long as the sequences matched partially. Structures with missing or multiple SCOP domain matches (a total of 11 proteins) were excluded in the following discussion.

**Fig. 6:**
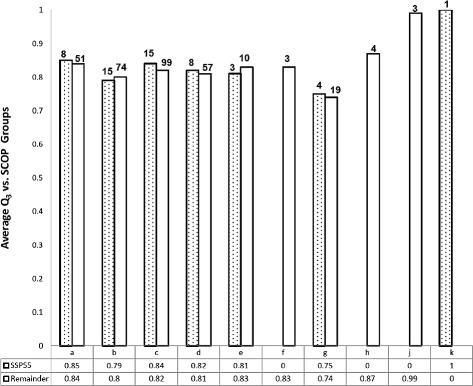
Q _3_ breakdown by SCOP classes **a**–**k**. Two types of Q _3_ are presented below the classes. 1. Tests on the SSP _55_ compact model proteins, which had been used in training (*shaded bars*). 2. Tests on the remainder of CB513 dataset NOT used in training (*white bars*). The Q _3_ for SSP _55_ is not necessarily higher than the remainder. Class g (*small proteins*) is the worst performing. A Q _3_ of 0 indicates no structures were found in that category (*absent bar*). The no. of structures present in each class is indicated above columns

The distribution of SCOP classes and Q _3_ scores in the compact model (SSP _55_) as well as the remainder of the CB513 dataset was compared (Fig 6). The results for SSP _55_ represent tests on the compact model itself. The 4 main protein structural classes according to SCOP are the (*a*) all alpha proteins, (*b*) all beta proteins, (*c*) interspersed alpha and beta proteins and (*d*) segregated alpha and beta proteins. Additional classes are (*e*) multi domain proteins for which homologues are unknown, (*f*) membrane and cell surface proteins, (*g*) small proteins, (*h*) coiled coil structures, (*j*) peptides and (*k*) designed proteins. Class (*i*) low resolution proteins, are absent from the dataset.

All the 4 main protein structural classes were found to have high Q _3_ scores ranging from 85 % for the alpha proteins (*a*) to 80 % for the beta proteins (*b*). The best performing proteins were those rich in Helix residues as expected (Class (*a*)). However, the lowest performing class was that of small proteins (*g*) with a Q _3_ of 74 % (averaged over 19 structures), rather than *β*-strand containing classes such as (*b*), (*c*), or (*d*) as might be inferred from the Sheet residues having the worst performance. One explanation is that poor Sheet performance arises from mispredicted single residue strands (state B of DSSP). These may be harder to predict than extended strands (state E of DSSP) which form more larger and more regular structures that are used in classifying proteins.

Additionally the prediction of Q _3_ is always much lower for Sheet structures since the hydrogen bonds are formed between residues that have high contact order; they are separated by many residues along a chain so these contacts are outside the sliding window. Hence, they are difficult to predict by sliding window-based methods. Also, the predictions are usually unreliable at the end of secondary structure elements. Thus, if there are many shorter secondary structures to be considered (such as for small proteins), the accuracy may be lower, which may account for the poor performance of small proteins (SCOP class (*g*)).

Overall there was hardly any difference in average Q _3_ scores between the compact model (SSP _55_) and testing proteins of CB513. Training a classifier with a given protein and subsequently testing the classifier on that same protein is expected to have a higher accuracy than if an unseen protein sample were presented to the classifier. However, for SCOP classes *a*, *g* and *c* the average Q _3_ of SSP _55_ was only marginally higher than the testing set at 1 % and 2 % respectively. This is an extremely small difference (1 % is approximately 11 residues in class *a* of SSP _55_). Unexpectedly, the Q _3_ of the testing proteins was higher in classes (*b*) and (*e*) instead. It is suggested that some intrinsic structural features of a protein arising from its *class*, pose a greater limitation on the predictive accuracy than if a given classifier has ‘learnt’ a particular protein (or class) previously. The confusion matrices of SSP _55_ and the remainder of the CB513 proteins broken down by their SCOP classes are available in Additional file 1: Tables S3 and S4, respectively.

### Blind tests of the compact model

The SSP _55_ and SSP _*CV*_ training models were tested in blind prediction experiments on a dataset of G Switch proteins (GSW25). Here the first and last four residues of the G Switch Proteins were included unlike the previous tests on CB513 (see *Development of compact model*). Although the training models did not include the first and last four residues of proteins, for a fair study, the normalization of the GSW25 proteins was done with respect to maxima and minima of the CB513 dataset that included the first and last four residues. For SSP _*CV*_, parameters from the best performing cross-validation partition were selected. Results are in Table 2.

**Table 2:** Results for G switch proteins (1400 residues)

Model	Observed *j*		Predicted *j*		Q _*j*_ (%)	Q _3_ (%)	SOV _*j*_ (%)	SOV (%)	MCC _*j*_
		H	E	C					
	H	682	1	39	94.46		100		0.79
SSP _*CV*_	E	58	352	136	64.47	76.65	66.04	59.07	0.65
	C	53	40	39	29.55		62.75		0.13
	H	680	0	42	94.19		100		0.83
SSP _55_	E	25	384	137	70.33	80.36	63.68	62.44	0.73
	C	51	20	61	46.22		78.91		0.25

SSP _55_ scored higher (Q _3_ = 80.36 %) than the conventional cross-validation model, SSP _*CV*_ (Q _3_ = 76.65 %). The widest difference was found for the Sheet and Coil classes, with Q _*E*_ and Q _*C*_ accuracies of SSP _55_ at 70.33 % and 46.22 % respectively, compared to much lower accuracies of 64.47 % and 29.55 % obtained by SSP _*CV*_ training. The SOV score was slightly higher for SSP _55_ at 62.44 % compared to 59.07 % of SSP _*CV*_.

Both training models achieved perfect SOV scores for the helix segments (SOV _*H*_ = 100 %), but difficulties arose for the Sheet and Coil predictions. The SSP _*CV*_ model was better than SSP _55_ for Sheet segment predictions (SOV _*E*_ of 66.04 % vs 63.68 %). However, there was a sharp drop in scores for the Coil residues (SOV _*C*_ = 78.91 % vs 62.75 %) for the former. The class-wise Matthew’s Correlation Coefficients (MCC) supported the results further. For MCC _*H*_, SSP _55_ obtained 0.83, vs 0.79 obtained by SSP _*CV*_, for MCC _*E*_, 0.73 vs 0.65 and for MCC _*C*_, 0.25 vs 0.13, respectively for each model. The SSP _55_ further had a better ability to distinguish between Helix and Sheet residues compared to the SSP _*CV*_ model; the helix to strand and vice versa mispredictions quantified by Q _*HEerror*_ are 1.8 % for SSP _55_ which were about two times lower as those obtained by SSP _*CV*_ at 4.2 %. The PDB structures of G Switch proteins (e.g. 2KDM) indicated that most of the Coil residues in the dataset are present at the ends of helical segments connecting one helix to another, which resulted in extremely low scores for this class. The Coil structures located at the end of structure segments are an area of future work. The compact model was further compared with several existing methods.

#### Comparison with other methods

The performance of SSP _55_ was compared with five well-known secondary structure prediction methods in the literature. These are the homology-based predictors SSpro [33] and PROTEUS [17] as well as the top-performing ab-initio predictors, PSIPRED [20], SPINEX [19] and PORTER [15]. These methods were recently assessed in a comprehensive survey in which they obtained Q _3_ accuracies between 80 to 82 % on a dataset of nearly 2000 protein chains [22]. Recent versions were used for three methods: PORTER 4.0 [58], PROTEUS 2 (http://www.proteus2.ca/proteus2/index.jsp) and a recently updated server for the SPINE method named SPIDER2, (http://sparks-lab.org/yueyang/server/SPIDER2/) that utilizes deep learning to predict several structural properties [59]. Results for FLOPRED, which used an extreme learning machine classifier employed with identical feature encoding data to those used in this work, have also been presented [37]. All results are in Table 3, ordered according to Q _3_. For consistency, all method names have been capitalized in the following discussion.

**Table 3:** Methods comparison on G Switch Proteins

Method	Observed *j*		Predicted *j*		Q _*j*_ (%)	Q _3_ (%)
		H	E	C		
	H	680	0	42	94.19	
SSP _55_	E	25	384	137	70.33	80.36
	C	51	20	61	46.22	
	H	665	19	38	92.11	
FLOPRED	E	41	380	125	69.6	78.72
	C	49	26	57	43.19	
	H	556	50	116	77.01	
PROTEUS 2	E	17	302	227	55.32	61.72
	C	2	124	6	4.55	
	H	519	99	104	71.89	
PSIPRED	E	167	243	136	44.51	57.36
	C	5	86	41	31.07	
	H	405	99	218	56.1	
PORTER 4.0	E	22	267	257	48.91	51.08
	C	0	89	43	32.58	
	H	473	95	154	65.52	
SPIDER2	E	112	213	221	39.02	50.79
	C	0	107	25	18.94	
	H	368	162	192	50.97	
SSPRO	E	13	312	221	57.15	50.43
	C	1	105	26	19.7	

The SSP _55_ compact model proved better than the 6 methods in predicting the secondary structure states of the G Switch proteins with a Q _3_ of 80.36 %. FLOPRED obtained the next best Q _3_ of 78.72 % followed by PROTEUS 2, PSIPRED, PORTER 4.0, SPIDER2 and SSPRO at 61.72 %, 57.36 %, 51.08 %, 50.79 % and 50.43 %, respectively. Unlike results for the CB513 dataset, the worst performing residues were coils rather than strands, with Q _*C*_ approaching 4.5 % for PROTEUS 2. Overall, Coil residues had been wrongly classified by most methods as Sheets with Q _*CE*_ (i.e. coils mispredicted as sheets) that ranged from 65 to 94 %. For the homology based methods SSPRO and PROTEUS 2 it is possible that wrongly assigned structural states from a high scoring but poor fitting template resulted in the low scores. In general, the remainder of the measures showed a poor performance for the Helix and Sheet classes, with the former being more successfully predicted for PSIPRED, PROTEUS 2 and PORTER 4.0. SSPRO however predicted the Sheet residues more successfully than the Helix residues.

Results from FLOPRED were similar to those of the SSP _55_ model, but the latter performed slightly better. The largest margin was for Coil with Q _*C*_ of SSP _55_ being 3.03 % higher than FLOPRED. For Sheet and Helix, FLOPRED scores were extremely close to those of SSP _55_.

The choice of feature encoding likely plays a role in the better results shown by SSP _55_ and FLOPRED since both have used energy based feature representation in comparison to other methods employing PSSM. The better results obtained by SSP _55_ over SSP _*CV*_ indicate that the choice of training proteins is highly important to preserve the generalization ability of the classifier and that, it is not necessary that a larger number of training proteins is a guarantee of good performance.

Here, energy based feature representation has been employed with a complex-valued neural network classifier. However, the derivation of a compact training model could potentially be used in subsequent works employing different classifiers or feature representation techniques. One important criteria for consideration is the speed of the learning algorithm. This should be sufficiently fast to produce results from large numbers of prediction trials, for selection of various training sets.

While the real-value neural networks may also be used in the derivation of the compact model, the FCRN shows a slightly better performance. Table 4 indicates that, for the G Switch Proteins dataset, the FCRN Q _3_ is slightly better than a 2-layered standard feed forward Multi Layer Perceptron (MLP) employing a conjugate gradient descent algorithm. Both the FCRN and MLP have been allowed 100 hidden neurons and are given exactly the same training samples. For the G Switch proteins the FCRN Q _3_ is higher by 1.14 %. This could be attributed to the extra decision boundary of the Complex plane employed in the FCRN hidden layer that enhances separability. For the same number of hidden neurons, the FCRN is slightly advantageous over the standard real networks.

**Table 4:** FCRN and MLP performance on G Switch Proteins

Method	Observed *j*		Predicted *j*		Q _*j*_ (%)	Q _3_ (%)
		H	E	C		
	H	680	0	42	94.19	
FCRN	E	25	384	137	70.33	80.36
	C	51	20	61	46.22	
	H	691	0	31	95.71	
MLP	E	38	394	114	72.17	79.22
	C	51	57	24	18.19	

Both networks were trained with SSP _55_

Some deficiencies of our technique are noted to be addressed in future works. First, the feature representation process is time consuming since reference energies must be computed across all templates (estimated at 2 hrs/100 residues on a 2.3 GHz processor with 8G RAM). Second, the poor Coil residue predictions (MCC _*C*_=0.25) for the GSW25 dataset leave much room for improvement.

In our earlier paper we had shown that we have removed possible similarities between proteins in the CB513 dataset and the CATH supplementary template structures, and therefore the performance of our method does not depend on significant homologies between these sets (See Supplementary Data in [37]). It is suggested that some theoretical support for the success in predictive accuracy in using a small set of training proteins is provided by work in protein fold space. In 2009, Skolnick et. al., demonstrated that protein fold space could be visualized as a continuum with each protein structure being related to another by 7 transitive structures, applied to single domain proteins at most 300 residues long [60]. Therefore, most structures are related and it is possible to “traverse” from one structure to another in fold space given some constraints such as the limits on domains or residue numbers. An efficient sampling of protein fold space results in some training sets being better than others. However, it is difficult to directly elucidate the structural relationship between train and test proteins that makes such performance possible; the inclusion of a certain protein fold in training does not directly give the classifier an ability to predict new structures similar to that fold.

### Case study of two inhibitors

Most of the errors in SS prediction arise from an inability of classifiers to distinguish between: (i) Sheet and Coil and (ii) Helix and Coil [18]. A comparison of two inhibitors in this section gives a possible reason for (i). Coil structures involved in hydrogen bonds with peptide backbone atoms were observed to be predicted as Sheet, while those preferring hydrogen bonds with waters were correctly predicted as Coil.

The worst performing sequence in the experiments conducted was the trypsin inhibitor molecule (PDB: 1MCT) with a Q _3_ of 40 % from the CB513 dataset. The predicted region of the inhibitor peptide was 20 residues (28 residues for entire peptide). Despite the small size, the molecule is of interest because none of the compared methods were able to achieve a Q _3_ greater than 60 %. The Q _3_ was poor even if the entire sequence was considered, or included in training. The accuracies of the methods for this sequence, in descending order were PORTER (60 %), PSIPRED (45 %), PROTEUS 2 (45 %), SSP _55_ (40 %) and SSPRO (30 %). Seventy percent of predicted residues adopt the Coil state and more than half of these were misclassified as Sheets by SSP _55_ (see Table 5). Likewise for other methods most of the errors were Coils misclassified as Sheet, or vice versa.

**Table 5:** Observed and predicted SS in two Inhibitors by SSP _55_

Trypsin Inhibitor, Q _*C*_ = 42.8 %																												
AA	R	I	C	P	**R**	**I**	**W**	**M**	E	C	T	R	D	S	D	C	M	A	**K**	**C**	**I**	C	V	**A**	G	H	C	G
OB					**C**	**C**	**C**	**C**	E	C	C	C	H	H	H	C	C	C	**C**	**C**	**C**	E	E	**C**				
PRED					**E**	**E**	**E**	**E**	E	C	C	C	C	C	C	C	C	C	**E**	**E**	**E**	E	C	**E**				
Kinase Inhibitor, Q _*C*_ = 100 %																												
AA	T	T	Y	A	D	F	I	A	S	G	R	T	G	R	R	N	A	I	H	D								
OB					H	H	H	C	C	C	C	C	C	C	C	C												
PRED					H	H	C	C	C	C	C	C	C	C	C	C												

Coil residues mispredicted as Sheets are in bold

**Fig. 7:**
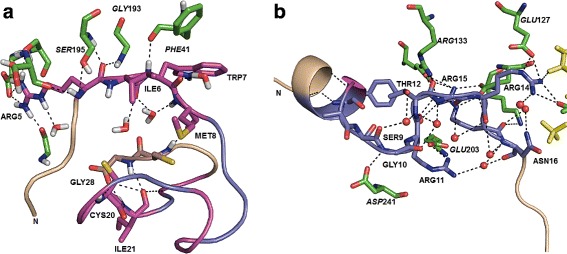
Detailed views of Coil prediction in inhibitors. **a** Porcine trypsin inhibitor (PDB entry: 1MCT). **b** cAMP dependent protein kinase inhibitor (PDB entry: 1ATP) with partially visible ATP in *yellow*. Correct predictions are in *light purple* and wrong predictions are in magenta. First and last four terminal residues are *light brown* and are not predicted. N marks the N-terminal. 1ATPI has more correct predictions than 1MCTI. Residues **RIWM** (5–8) and **KCI** (19–21) of 1MCTI are Coils wrongly predicted as Sheets. Residues **ASGRTGRRN** (8–16) of 1ATPI are correct Coil predictions. Waters are *red and white sticks* in **a** and *red spheres* in **b**. Putative hydrogen bonds (h-bonds) are indicated with *dashed black lines*, identified by inhibitor polar atom centres within 3.6Å of any O, N atoms. *Italics* denote the respective enzyme residues (*green*). The trypsin inhibitor residues make several h-bonds with peptide backbone O, N atoms and the kinase inhibitor, none. Examples in **a** ARG5 CO with *GLY*193 NH; ILE6 NH with *PHE*41 CO. The kinase inhibitor prefers side-chain and water molecule contacts. Examples in **b** SER9 N with *ASP*241 OD1; THR12 CO with *ARG*133 NH1; ARG14 CO with two waters. Not all h-bonds are shown; see text for more

The methods compared differed in factors such as feature encoding, learning algorithm and underlying training models. Most have likely already included the trypsin inhibitor as part of training since it belongs to an older dataset. The persistent poor predictions could therefore arise from structural features that remain difficult to capture by current techniques. To characterize the structural environments that are a source of mistakes between Coil and Sheet classes, comparisons were made with the peptide inhibitor of the cAMP dependent protein kinase (PDB: 1ATP). The kinase inhibitor was of a comparable length (20 residues, of which 12 were predicted) and comprises 75 % Coil in the predicted region. Unlike in the trypsin inhibitor, all observed Coils are predicted correctly by SSP _55_ (Q _*C*_ = 100 %). The Q _*C*_ of other methods were PORTER (100 %), PSIPRED (88.9 %), PROTEUS 2 (100 %) and SSPRO (88.9 %). The inhibitor sequences and their observed and predicted SS states by SSP _55_ have been presented in Table 5. Both inhibitors appear to comprise mostly of long loop regions with the kinase inhibitor possessing a 7-residue long N-terminal helical segment followed by a 13 residue Coil segment (see Fig 7b).

In the trypsin inhibitor, the peptide segment ’RIWM’ (residues 5–8) and ’KCI’ (residues 19–21) were Coils that had been wrongly predicted as Sheets. CYS20 and ILE21 in particular, were wrongly predicted as Sheets in all methods tested. In the kinase inhibitor, the 9 residue coil segment ’ASGRTGRRN’ (residues 8–16) was predicted correctly as Coils. Coil regions from both molecules are involved in extensive hydrogen bonds with their respective enzymes and water molecules. However, an important difference is that the trypsin inhibitor participates more heavily in hydrogen bonds formed by carbonyl oxygen (CO) or amide NH groups of the peptide backbone (either the trypsin molecule, or its own peptide segments that are turned upon itself). In contrast, the kinase inhibitor relies more on hydrogen bonding with water molecules to maintain the complex (Fig 7).

#### Detailed hydrogen bonded contacts

The putative hydrogen bonds listed in the discussion below are inferred from distance based polar contacts using PyMOL (http://www.pymol.org/). Capitalised italics indicate residues from the trypsin and protein kinase chains in their respective complexes. Numbers following three letter amino acid abbreviations correspond to residue numbers of ATOM records in their respective PDB files.

##### Trypsin inhibitor:

Bonds involving peptide backbone atoms are listed for this inhibitor (PDB: 1MCTI. Figure 7a shows some of these). The carbonyl oxygen (CO) of ARG5 in bifurcated hydrogen bonds with the amide (NH) of *SER195* and *GLY193*; NH of ARG5, hydrogen bonded with CO of *SER195*; NH of TRP7 with CO of *PHE41*; CO of MET8 with NH of CYS27; NH of LYS19 with CO of ILE2; CO of ILE21, with NH of GLY28; NH of CYS20, is hydrogen bonded to CO of MET17 and so forth. Besides these, several potential contacts with water molecules are seen; CO of ILE6 which participates in bifurcated hydrogen bonds with 2 waters, CO of TRP7, NH of MET8, NH of MET17 and CO of CYS22 all of which participate in hydrogen bonds with one water molecule, each [61].

##### Kinase inhibitor:

For this inhibitor (PDB: 1ATPI), only one hydrogen bond involving the peptide backbone, NH of SER13 with CO of PHE10, is observed. Apart from SER13, no others in residues 8–16 are observed to potentially contain hydrogen bonds involving the peptide backbone (CO …HN), although sidechain contacts such as (GLY10 N and *ASP241* OD) are possible. Instead, water molecules are observed to be in contact, such as: SER9 CO, GLY10 CO, THR12 N, ARG14 CO, ARG15 CO and so forth with nearby waters (see Fig. 7b for examples). Not all putative hydrogen bonded contacts are listed.

Not all wrongly predicted Coils may be attributed to the presence of hydrogen bonding involving the peptide backbone. For instance in 1MCTI, CO of Sheet residue VAL23 is hydrogen bonded to HIS26 N and is wrongly predicted as Coil. However it is possible to infer from the structural comparisons that the kinase inhibitor relies more heavily on water mediated hydrogen bonds than does the trypsin inhibitor.

The solvent accessibilities of individual residues in both predicted segments of the inhibitor peptides, as well as the hydrophobicity of residues were considered. However, it was difficult to distinguish the differing Q _*C*_ accuracies based on these characteristics. The crystal structure resolutions are 1.6 Å and 2.2 Å for 1MCT and 1ATP respectively. If low resolution were a factor the prediction for the kinase inhibitor (PDB: 1ATP) should be of poorer quality, but the opposite is observed. The effect of hydrogen bonds contacts (whether between main-chains to involving waters) on residue misprediction is further investigated by analysing all structures in the CB513 dataset.

In the following discussion, hydrogen bond contacts of protein main-chain atoms are investigated. In particular, the proportion of contacts formed between main-chain atoms and water atoms in correct vs. mispredicted residues, is discussed. When the entire dataset is considered, evidence suggests that the presence of water-mediated hydrogen bonding can influence misprediction rates. In particular, the type of hydrogen bond contacts a residue makes- whether only between main chain atoms, or involving water molecule, is a factor.

The HBPLUS software [62] was used to detect putative hydrogen bonds in the 385 chains of the CB513 dataset. Nine chains had to be discarded from the analysis, since their PDB derived sequences did not match their CB513 sequences. The **D**onor-**A**cceptor (DA) distance, specifies the maximum allowed distance between the hydrogen-bond donor and acceptor atoms. The DA distance was set to 3.6Å and other settings were the default values.

The results of the case study indicated that for mispredicted Coils, the main chain atoms are more likely to be in contact with other main chain atoms. Conversely, the correctly predicted Coils were more likely to be in contact with hetero-atom water molecules. The notation of HBPLUS was followed. Here, the Donor (D) or Acceptor (A) role is ignored; as long as a (M)ain chain atom of a residue satisfies hydrogen bonding geometry with any other (M)ain chain atom, the bond is denoted as MM. If the main chain atom forms a potential contact with water (H)etero-atom in the structure, the bond is classified as MH. Therefore MM denotes two main chain atoms that act as DA, while MH denotes a main chain atom and (water) hetero-atom that are DA. The MM and MH counts are presented in Table 6.

**Table 6:** Detected hydrogen bonds of sheet and coil residues

	MM	MH	MM + MH	$\frac {MM}{(MM+MH)}$ (%)	$\frac {MH}{(MM+MH)}$ (%)	All	No. of residues
R _*CC*_	10345	14690	25035	41.3	58.7	78700	19182
R _*CE*_	1685	1898	3583	47.0	53.0	10652	2584
R _*EC*_	3972	2303	6275	63.3	36.7	17052	3193
R _*EE*_	15143	5732	20875	72.5	27.5	51286	10370

Types of hydrogen bond contacts considered are from **M**ain-chain to **M**ain-chain (MM) atoms and **M**ain-chain to **H**etero-atom Water (MH) atoms. MM + MH is their sum. All indicates all hydrogen bonds including those involving side chains. R _*ij*_ denotes a residue in native state *i* predicted as *j*

For Coils mispredicted as Sheets (R _*CE*_), the rate of participation in main-chain to main-chain hydrogen bond contacts (MM) is 47 % compared to that of correctly predicted Coils (R _*CC*_), 41.3 %. Correctly predicted Coils also have a higher rate of main-chain to water molecule hydrogen bond contacts (MH) compared to those mispredicted as Sheets (58.7 % vs 53.0 %). For Sheet residues, the distinction between the proportion of MM and MH contacts, is more apparent. For correctly predicted Sheet residues (R _*EE*_), 72.5 % of main chain atom contacts are with other main chain atoms when compared against a total of main-chain to main-chain and main-chain to water contacts (MM+MH). Main-chain to water atom contacts (MH) comprise the remaining 27.5 %. For Sheet residues mispredicted as Coil (R _*EC*_), the proportion of main-chain atoms involved hydrogen bonded contacts with water molecules, is higher at 36.7 %.

The implications of these findings are discussed. Since regular, hydrogen bonded geometry of the peptide backbone forms the major definition of the secondary structure states, main-chain atoms that are in potential hydrogen bonds with water atoms could be harder to predict correctly, for the Sheet residues. For the Coil residues, having more contacts with water atoms (and therefore, less with the nearby main-chain atoms) results in them having a higher chance of being predicted correctly rather than being misclassified as Sheet. The other types of contacts made, such as towards non-water hetero-atoms and also to Side-Chain atoms, are not discussed here, but the total number of all hydrogen bonded contacts made, as well as the number of residues for which the hydrogen bond counts were made, is provided in the Table 6.

From the structures, it is suggested that residue segments in flexible or coil like states which participate in hydrogen bonding with peptide backbone atoms of spatially close residues may be misclassified as Sheets, since such type of bonding is similar to the peptide backbone hydrogen bonding commonly found in Sheets. However, residue segments in loop or Coil conformation that participate in extensive water coordination could be predicted with greater ease. This is in agreement with previous findings that solvent exposed coils are predicted with greater accuracy than buried coils, since buried coils are more likely to interact with other protein atoms [22].

Unlike the energy based CABS encoding, the PSSM based feature representation contains no structure comparison steps that could be an indirect source of structure-based information. Nevertheless, methods employing both types of feature encoding techniques, failed to capture the trypsin inhibitor adequately. It therefore, is possible that the ambiguity between Sheet and Coil classes in mispredicted residues arises at the level of secondary structure detection and assignment, due to the environment of main-chain atoms. For instance, a Sheet residue’s main-chain CO in proximity to a water molecule, has another potential hydrogen bond Donor, rather than only the NH group in a typical hydrogen bonded *β*-sheet geometry. This could in turn be harder to predict, than if the water molecule were absent. The findings of Table 6 suggest that mispredicted Sheet residues have a higher proportion of water molecule contacts than correctly predicted Sheets.

Previous works sought to investigate the residue contact order and to increase the sliding window sizes to accommodate long-range interactions. Another factor that may be responsible for persistently poor prediction (such as the inhibitor peptide discussed) is the role of the structural environment of the protein main-chain atoms in the mis-prediction rates. This could assist the improvement of future secondary structure prediction methods and has not been considered before.

A difficulty of distinguishing between Coil residues involved in hydrogen bonds with the peptide backbone and Sheet residues was identified in this work. This is reflected in the higher accuracies for the kinase inhibitor as compared to the trypsin inhibitor across all methods compared, despite both peptides comprising largely of Coils.

## Conclusions

In conclusion, the choice of training proteins can affect the classifier performance. Results from employing the compact model for secondary structure prediction indicate that training classifiers on large numbers of proteins may lead to loss of prediction ability when faced with new sequences. This hints at the presence of structural relationships between train and test proteins that may influence prediction results.

In general, a compact model has two practical advantages which are the small size allowing rapid training and more importantly, a good preservation of the classifier’s generalization ability. At the same time, the secondary structure preferences seen in the large data sets are encoded in the context-dependent statistical potentials of the CABS force-field used in our method, thereby making the secondary structure predictions less dependent on the training set.

The case studies presented highlight the difficulty of current secondary structure prediction techniques in handling some chains, even if they were to be included in the dataset of the training proteins.

Specifically, Coil residues of the trypsin inhibitor that contained hydrogen bonding involving the peptide backbone atoms were found to have been predicted as Sheet. Conversely, Coil residues of a protein kinase inhibitor (of similar length) had been correctly predicted, with the structural difference being that these were involved in an extensive water-mediated hydrogen bonding network that maintained the complex. This highlights the possible need for methods that can accurately distinguish between Sheet and Coil residues involved in different types of hydrogen bonding. Other limits of the current approach that need to be addressed in future work are, the reduction of time taken for the CABS-algorithm based feature encoding process as well as an automated procedure that can locate the key proteins to be included in training for any given dataset.

## Additional file

Additional file 1
**Table S1.** The 25 sequences of the G Switch Proteins dataset (GSW25). The 12 G _*A*_ sequences and 13 G _*B*_ sequences are given and cited with their original source. **Table S2.** The 55 proteins of the compact model (SSP _55_). The protein names, SCOP classes, folds, number of residues, and the Q _3_ achieved per protein are given. **Table S3.** The confusion matrices broken down by SCOP classes, are given for the SSP _55_ proteins. **Table S4.** The confusion matrices broken down by SCOP classes, are given for the remainder of the CB513 dataset (330 proteins). (XLSX 30 KB)

## Abbreviations

SS: Secondary structure
SSP: Secondary structure prediction
SCOP: Structural classification of proteins
FCRN: Fully complex-valued relaxation network
CABS: C-Alpha, C-Beta, Side-chain
SSP _55_: Secondary structure prediction with 55 training proteins (compact model)
SSP _*CV*_: Secondary structure prediction by cross-validation
